# An In Silico Framework to Mine Bioactive Peptides from Annotated Proteomes: A Case Study on Pancreatic Alpha Amylase Inhibitory Peptides from Algae and Cyanobacteria

**DOI:** 10.3390/nu14214680

**Published:** 2022-11-04

**Authors:** Lorenzo Pedroni, Florinda Perugino, Gianni Galaverna, Chiara Dall’Asta, Luca Dellafiora

**Affiliations:** Department of Food and Drug, University of Parma, 43124 Parma, Italy

**Keywords:** bioactive peptides, pancreatic alpha amylase inhibitors, anti-nutrients, bioinformatics, molecular modeling, in silico digestion

## Abstract

Bioactive peptides may exert beneficial activities in living organisms such as the regulation of glucose metabolism through the inhibition of alpha amylases. Algae and cyanobacteria are gaining a growing interest for their health-promoting properties, and possible effects on glucose metabolism have been described, although the underlying mechanisms need clarification. This study proposes a computer-driven workflow for a proteome-wide mining of alpha amylase inhibitory peptides from the proteome of *Chlorella vulgaris*, *Auxenochlorella protothecoides* and *Aphanizomenon flos-aquae*. Overall, this work presents an innovative and versatile approach to support the identification of bioactive peptides in annotated proteomes. The study: (i) highlighted the presence of alpha amylase inhibitory peptides within the proteomes under investigation (including ELS, which is among the most potent inhibitory tripeptides identified so far); (ii) mechanistically investigated the possible mechanisms of action; and (iii) prioritized further dedicated investigation on the proteome of *C. vulgaris* and *A. flos-aquae*, and on CSSL and PGG sequences.

## 1. Introduction

Bioactive peptides are short amino acid sequences (from two to twenty residues) exerting biological activity in living organisms once they are released from the protein in which they are encrypted [1]. They cover a broad spectrum of presumed biological properties including anti-hypertensive actions, antioxidant effects, regulation of cholesterol level and glucose metabolism [2], although their actual in vivo activity still lacks a consensus. Concerning the capability to regulate glucose metabolism, the inhibition of pancreatic alpha amylase is among the key early mechanisms underpinning the possible hypoglycemic properties of bioactive peptides [3]. Specifically, the inhibition of alpha amylases can impair the overall metabolism, eventually affecting the growth of animals when inhibitors are potent and given at high levels. However, inhibitory peptides have also been described as beneficial when used to co-treat obesity or diabetes [4,5,6]. Therefore, the identification of alpha amylase inhibitory peptides in food is fundamental both to better understand the effects of a given food on human health and to rationally design functional foods, ingredients, and targeted nutritional interventions.

Nowadays, the identification and characterization of bioactive sequences in food is still costly and time-consuming as it is based on high-throughput methods coupling advanced analytical approaches and biological assays [7,8]. However, in silico and bioinformatics methodologies have been widely used in the last decade, together or as stand-alone techniques, to support the high-throughput analysis of proteins and derived peptides [9,10,11,12,13]. These approaches may succeed in mining bioactive peptides of food origin supporting the straightforward and high-throughput identification of sequences active over a variety of targets and systems [14,15,16], also as stand-alone techniques [17,18,19,20]. In this context, the present work aimed at developing a computational workflow integrating bioinformatics and molecular modeling to mine pancreatic alpha amylase inhibitory peptides from the annotated proteome of *Aphanizomenon flos-aquae* (AFA; Klamath algae)*, Chlorella vulgaris* and *Auxenochlorella protothecoides*. Based on their presumed beneficial effects on living organisms, the cyanobacteria AFA and the two microalgae *C. vulgaris* and *A. protothecoides* have a long history as a food source or as a source of nutraceuticals and functional ingredients for food and feed [21,22]. Particularly, potential antidiabetic properties have been described [23,24,25], although the comprehension of the underlying mechanisms and the profiling of the bioactive chemical complex are still far from being clarified. Based on their high protein content (up to 70% of algal biomass) [21,26], it is likely that algae and cyanobacteria also contain alpha amylase inhibitory peptides with possible consequences on the modulation of glucose metabolism and bioavailability. However, despite the intensive research carried out in the past years, this specific aspect is still largely overlooked, preventing a full understanding of the mechanistic connections between microalgae’s macro-nutrients (protein in this case) and their possible effects in living organisms.

In this context, this work presents an innovative and effective computational workflow to study the bioactivity of peptides encrypted in a selection of annotated algal proteomes and shows that the proteome of cyanobacteria and microalgae included in this study may contain and release peptides with previously demonstrated alpha inhibitory activity. The presence of novel inhibitory sequences never described before are highlighted for further dedicated studies.

## 2. Materials and Methods

### 2.1. Bioactive Peptide Analysis 

#### 2.1.1. Data Retrieval

The list of sequences annotated as alpha amylase inhibitory peptides analyzed in this study was defined based on an in-depth literature search performed browsing PubMed and Scopus database (last database accessed on 22 February 2022). To do so, the following search string was used: “amylase inhib*” AND “bioactive peptides”. The literature search was limited to papers published from 2012 to 2021 focusing on title, abstract and keywords fields. The set of papers retrieved (*n* = 44) was visually inspected to collect the relevant information concerning the bioactive sequences described, such as the primary protein sequence of peptides, the peptide activity (e.g., IC_50_) and the respective protein source. Nine papers (Table S1), and a total of 179 sequences (from two to eleven residues long) annotated as alpha inhibitory peptides were identified. Then, the analysis focused on a subset of tri- and tetra-peptide (the underlying rationale is detailed in Section 3.1). All the sequences were converted in the FASTA format for further analysis. 

All the available proteins of *C. vulgaris*, *A. protothecoides* and AFA (including the 2012/KM1/D3 strain) were retrieved from UniProtKB (https://www.uniprot.org) [27] filling up the Advanced Search Organism (OS IDs 3077, 3075, 1176 and 1532906, respectively; last database accessed on 22 February 2022).

#### 2.1.2. Searching Bioactive Peptides within Algae and Cyanobacteria Proteome

An iterative local sequence alignment procedure was set up based on the Smith-Waterman algorithm (EMBOSS 6.6.0.0) [28] and an in-house bash script was written to iteratively search each bioactive peptide sequence within the selected proteomes. The opening and extension gap penalty were set at 10 and 0.5, respectively. Then, each output alignment was concatenated in a unique file used as input for an in-house python script able to parse it and to render it as an easily readable table for further data processing (see Table S2 for further details).

### 2.2. Molecular Modelling 

#### 2.2.1. Peptides and Protein Model Design

The 3D structures of tripeptides analyzed in this study were generated in the .mol2 format using the Builder Protein tool implemented in PyMol (version 2.3.0), setting the C- and N-terminal as deprotonated and protonated, respectively. The model for porcine alpha amylase was derived from the crystallographic structure with PDB code 1HX0 [29], retrieved from the Protein Data Bank (https://www.rcsb.org; last database accessed on 4 May 2022), removing water and the other co-crystallized ligands before running the analysis. 

#### 2.2.2. Pocket Scan and Docking Simulations

In agreement with a previous study [5], the identification of allosteric pockets, in addition to the substrate binding site, was performed using the GetCleft algorithm [30] with default parameters and setting the maximum number of pockets to identify at five. The peptides were then docked in each of the five identified pockets (arbitrarily centering the space to explore in a 10 Å radius sphere around the centroid of the pocket) allowing the most probable interaction site to be hypothesized. Docking simulations were performed using the GOLD software (Genetic Optimization for Ligand Docking, version 2021.10) in agreement with previous studies [15,31] using the internal PLPScore function to estimate the fitting of each peptide within the pockets identified (the higher the score, the higher the expected peptide-pocket fitting). A semi-flexible docking approach was applied while allowing protein’s polar hydrogens free to rotate and considering peptides fully flexible. 

#### 2.2.3. Molecular Dynamic Simulations 

Molecular dynamic simulations were used to estimate the stability of protein–peptide complexes, in agreement with a previous study [15], using GROMACS (version 2019.4) [32]. The input complex structures were solvated with SPC/E waters in a dodecahedron periodic boundary condition and neutralizing the system with counter ions (Na^+^ or Cl^−^). Next, each system underwent an energetical minimization to avoid steric clashes and to correct improper geometries using the steepest descent algorithm with a maximum of 5000 steps. Then, all the systems underwent isothermal (300 K, coupling time 2 picoseconds) and isobaric (1 bar, coupling time 2 picoseconds) 100-picosecond simulations before undergoing 20-nanosecond simulations each (300 K with a coupling time of 0.1 picosecond and 1 bar with a coupling time of 2.0 picoseconds).

### 2.3. In Silico Digestion 

An in silico protein digestion analysis was performed using the python-based Rapid Peptides Generator (RPG, version 1.2.4) tool [33]. This tool requires protein FASTA sequence as input and accurately predicts protease-dependent and chemical means-induced cleavage sites allowing simulation of multiple protease digestion/hydrolysis for a precise estimate of peptide release. A concurrent and systematic digestion method was chosen, allowing all the possible binary combinations of enzymes and chemical means to digest the protein of interest. At the time of analysis (as of July 2022), the software implemented 42 enzymes/chemicals and an in-house python script was exploited to generate all the possible paired conditions (1722 combinations in total).

## 3. Results and Discussion

### 3.1. Data Retrieval 

The in-depth literature search identified nine papers useful for the sake of this study (Table S1). The papers were published between 2012 and 2021, and a total of 179 sequences (from two to eleven residues long) annotated as alpha inhibitory peptides were found. The analysis was then focused on tri- and tetra-peptides (34 sequences). The rationale underlying this choice was to focus the analysis on sequences with a high chance of being active per se in a real-world scenario. Indeed, longer (oligo)peptides proved to have a relatively low transepithelial permeability and they are likely to undergo proteolytic cleavage by brush boarder’s proteases [34]. Conversely, shorter sequences (di-, tri- and tetra-peptides) may have a higher epithelial permeability and bioavailability [35]. Table 1 reports the set of 34 alpha amylase’s inhibitory tri- and tetra-peptides initially meant to be searched within the algal proteomes under investigation. Only sequences unambiguously associated with an individual IC_50_ value were carried forth to the analysis (six sequences in total) while excluding those assessed in multi-peptide mixtures and lacking individual activity data. Of note, dipeptides were excluded from the analysis as they were only assessed in mixtures.

Afterward, all the protein sequences available for *C. vulgaris*, *A. protothecoides* and AFA were searched. To do so, the gold benchmark database of protein sequences, UniProtKB (https://www.uniprot.org) [27], was browsed. All the protein sequences were retrieved in the FASTA format grouping 596, 20,903 and 13,584 sequences for each organism, respectively (35,083 sequences in total; last database accessed on 22 February 2022). Notably, only 0.3% of them (103 out of 35,083 sequences) were annotated as “reviewed” protein and nearly 90% of them belonged to *C. vulgaris* (90 out of 103 sequences). The “reviewed” specification indicates the protein has been manually annotated based on transcription/expression information extracted from the literature or curator-evaluated computational analysis. Conversely, “unreviewed” specification indicates the protein has been computationally analyzed and automatically annotated, awaiting manual curation. Keeping in mind that this work aimed to identify actual protein sources of bioactive peptides, only the reviewed sequences were analyzed. 

From a general perspective, this evidence highlighted the scarce understanding and characterization of the algal proteomes under analysis. This is of particular concern considering the growing interest in algae either as an innovative, multi-purpose and valuable source of protein or for their use in food and feed production. Specifically, proteins may encrypt a vast variety of bioactive sequences, including but not limited to health promoting peptides. Indeed, short peptides with toxic or antinutritional effects have been reported too [36], and their identification in matrices meant to be used in food/feed production or as source of bioactive peptides is strongly advisable. In this context, the use of bioinformatics analysis may substantially support the study of bioactive peptides from food protein [37] while its usage to search bioactive peptides from structured database has been already reported [38]. However, the degree of characterization of the space under analysis, which is represented by the level of characterization of algal proteomes in this case, may relevantly affect the quality, the confidence, and the throughput of the analysis. In this respect, our study highlighted the very limited characterization of algal proteomes posing a compelling line of evidence for the urgent need of annotation to: (i) better understand the encrypted bioactive components; (ii) ensure a more informed use of algae and cyanobacteria in food and feed; and (iii) allow a more proficient use of bioinformatics.

### 3.2. Mining of Bioactive Sequences into Algal Proteomes

The in-depth literature search of tri- and tetra-peptides with inhibitory activity against pancreatic alpha amylase meant to be searched into algal proteomes identified 34 sequences (Table 1). However, 28 sequences were not considered for subsequent analysis as they were assessed in mixtures with only mixture activities reported with no information about their individual activity. Conversely, the six sequences tested as single substances and with an individual IC_50_ reported (namely CSSV, YSFR, SAAP, PGGP, ELS and GGSK) were further analyzed and searched in the reviewed fraction of algal proteomes under investigation. 

The search was performed using an iterative bioinformatic approach (see Section 2 for further details) to check the presence of CSSV, YSFR, SAAP, PGGP, ELS and GGSK within the list of reviewed proteins of *C. vulgaris*, *A. protothecoides* and AFA (Table S3). Interestingly, only ELS was found to occur in the set of reviewed algal proteins. Specifically, it was found in seven proteins of *C. vulgaris* and in one protein of AFA (Table 2). Notably, ELS is the most potent peptide in the list of those considered in this analysis (Table 1). The results collected highlighted a multiple occurrence of ELS in the reviewed fraction of *C. vulgaris* proteome and some of the containing proteins had homologues in the proteome of the *Porphyra* spp. where ELS was found and studied for the first time (Table 2). This evidence further pointed to the possible relevance of *C. vulgaris* proteins as a possible and relevant source of ELS, likewise *Porphyra* spp. Concerning AFA, ELS was found only in one protein (UniProt entry A0A0B0QJN8). The single occurrence among AFA proteins was likely due to the lower number of reviewed proteins compared to *C. vulgaris* and therefore AFA could also be considered as a possible source of ELS worthy of further dedicated investigations. Moreover, the missed identification of ELS within the proteins of *A. protothecoides* could be due to the very scarce annotation of its proteome, which included only six reviewed proteins. Therefore, its relevance as a possible source of ELS could not be excluded completely. 

Afterward, as a proof of principle, an additional sequence analysis was performed searching the presence of sequences similar to CSSV, YSFR, SAAP, PGGP and GGSK in the AFA’s phycocyanin. The similarity threshold was arbitrarily set at 75% of identity, allowing the subsequent analysis sequences to carry forth with only one residue substitution/removal. Based on the assumption that a single-residue substitution/removal may have a limited impact on peptide bioactivity, this choice was made to increase the chance of identifying novel sequences with a high inhibitory potential. This analysis targeted the AFA’s phycocyanin as it is the most abundant among the class of phycobiliproteins, which account for nearly 60% of the whole cyanobacteria protein content. The analysis revealed the presence of PGG, PGGN and CSSL in the C-phycocyanin beta subunit (UniProt entry P85869; residues 59–61, 59–62 and 116–119, respectively). PGG and CSSL were highly similar to their orthologous sequence (PGGP and CSSV, respectively) and they were deemed likely to retain a certain degree of activity. Therefore, they were considered for the subsequent analysis. Conversely, PGGN was not considered in the light of the substantial physico-chemical difference between N and G, which was expected to have a major impact on peptide bioactivity.

**Table 1:** List of tri- and tetra-peptides with reported inhibitory activity against pancreatic alpha amylase.

Sequence	Activity	Source	References
FLS	225 μg/mL ^a^	Yellow field pea	[39]
YAL			
TVF			
IFS			
FSL			
ERA			
EAR			
NKN			
KNN			
NNK			
PHY			
WNP			
GKGN			
SLSD			
VVSE			
TFPG			
ASFP			
IARP			
LQRF			
RVLD			
VDRI			
INKQ			
KQVQ			
DLRV			
VDRL			
IVDR			
KFFE			
ACGP	2.74 mM ^b^	Bovine casein	[40]
**CSSV**	34.88 mM ^c^	Chinese giant salamander (*Andrias davidianus*)	[41]
**YSFR**	18.93 mM ^c^		
**SAAP**	12.95 mM ^c^		
**PGGP**	12.96 mM ^c^		
**ELS**	2.58 ± 0.08 mM ^c^	Red seaweed (*Porphyra* spp.)	[42]
**GGSK**	2.62 ± 0.05 mM ^c^		

Note: ^a^ Peptide concentration with the highest inhibitory activity reported; ^b^ the reported activity (IC_50_) refers to the activity of a mixture of peptides containing ACGP; the activity of ACGP could be inferred accordingly; ^c^ Individual IC_50_; The sequences tested as a single substance with an individual IC_50_ reported are shown in bold.

**Table 2:** List of *C. vulgaris* and AFA reviewed proteins containing ELS.

Protein Name	UniProt AC	Corresponding Protein in *Porphyra* spp. Containing ELS
Translation initiation factor IF-1, chloroplastic ^a^	P56290	Homolog not detected
Photosistem II protein D1 ^a^	P56318	Photosistem II protein D1 (P51212)
ATP-dependent zinc metalloprotease FtsH homolog ^a^	P56369	Sequence not detected
ATP syntase subunit beta, chloroplastic ^a^	P32978	ATP synthase subunit beta, chloroplastic (P51259)
DNA direct RNA polymerase subunit alpha ^a^	P56298	DNA direct RNA polymerase subunit alpha (P51293)
Probable sulfate/thiosulfate import ATP-binding protein CysA ^a^	P56344	Homolog not detected
Photosystem I assembly protein Ycf4 ^a^	P56312	Photosystem I assembly protein Ycf3 (P51258)
Protein adenylyltransferase MntA ^b^	A0A0B0QJN8	Homolog not detected

Note: ^a^ indicates proteins of *C. vulgaris*; ^b^ indicates a protein of AFA.

### 3.3. Molecular Modeling Results

The subsequent analysis comprised a molecular modeling procedure to study the capability of PGG and CSSL to interact with the pancreatic alpha amylase to predict their inhibitory potential. To do so, the mechanism of inhibition of the orthologous inhibitory peptides PGGP and CSSV was studied first. In this respect, the mechanism of inhibition of alpha amylase inhibitory peptides was rarely investigated in the studies available in the literature at the time of analysis. As an example, for the set of sequences considered here (namely, CSSV, YSFR, SAAP, PGGP and GGSK), GGSK was only reported as a non-competitive inhibitor, while the mechanism was not reported for the other peptides. For this reason, GGSK was also included in the molecular modeling study along with CSSV and PGGP as a reference sequence to test the procedural reliability (see below).

**Figure 1:**
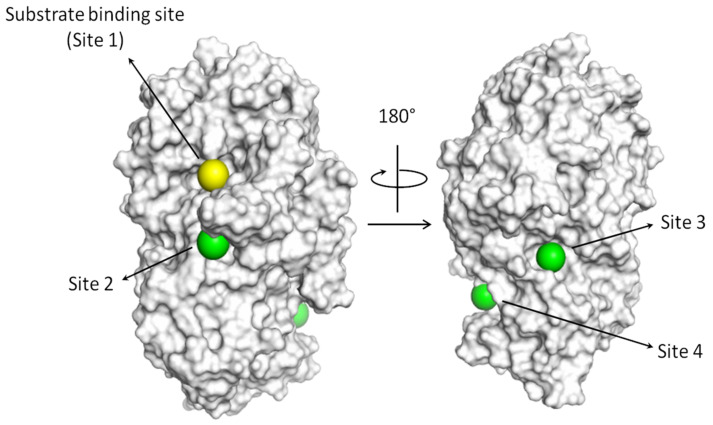
Surface representation of pancreatic alpha amylase. The yellow sphere indicates the location of the substrate binding site, while the green spheres indicate the location of the additional surface pockets considered in this study.

**Figure 2:**
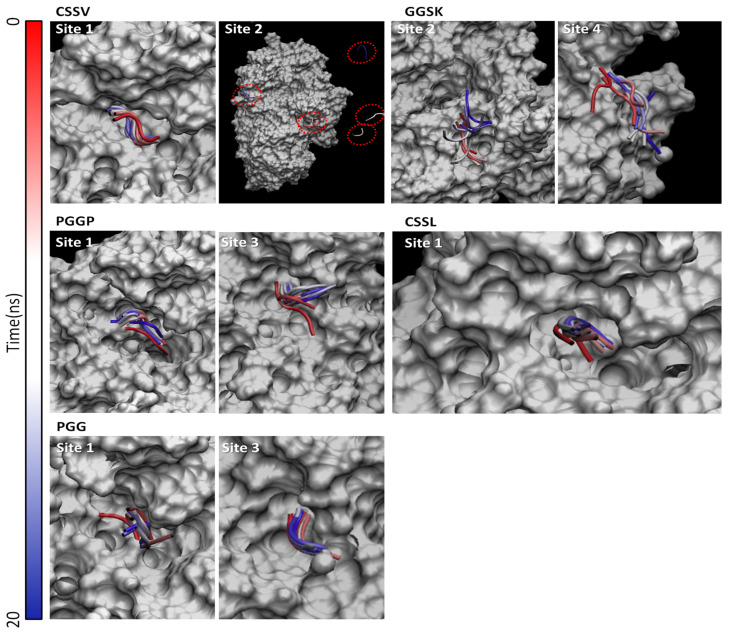
Trajectories of peptides under investigation. The protein is represented as white surface, while peptides are represented as tubes. The red-to-blue color switch indicates the stepwise changes of coordinates over time. The red dashed circles indicate the detachment of CSSV from site two.

The mechanisms of inhibition of pancreatic alpha amylase still need clarification. It has been proved that the competition with the substrate within the catalytic binding site may efficiently inhibit the enzyme, but the presence of allosteric site(s) has also been described [5]. However, the location of allosteric site(s) on the protein structure is not precisely identified yet. Indeed, previous computational studies identified up to seven different pockets, in addition to the substrate binding site, that could be targeted to inhibit enzymatic activity [5]. Following a similar approach, this study screened the protein surface identifying three major pockets in terms of dimension, in addition to the substrate binding site. Then, CSSV, PGGP and GGSK were docked within each surface pocket and within the substrate binding site (Figure 1). The most probable site(s) of interaction was identified based on the docking scores considering that the score amount is proportional to the physico-chemical match between the pocket and the various peptides (the higher the score, the more favorable the interaction within the given pocket), as previously reported [43]. As shown in Table 3, CSSV, PGGP and GGSK recorded comparably high scores in two different sites each (CSSV within site one and two; GGSK site two and four; PGGP site one and three) pointing to the possible multi-site interaction for all of them. The geometrical stability of the best scored protein–peptide complexes and the capability of peptides to persist at the identified binding sites over time was further assessed with molecular dynamic simulations. As shown in Figure 2, the analysis of peptide trajectories revealed that CSSV was found to stably interact with site one (i.e., the substrate binding site) but not with site two, GGSK was found comparably able to persist at site two and four, while PGGP was found to interact with site one and site three. On this basis, it was inferred that CSSV was likely to have a competitive mechanism of inhibition, while PGGP was likely to have a mixed mechanism as it was theoretically able to interact with site one (the substrate binding site) and three. Of note, the procedure excluded a competitive mechanism for GGSK as it was not found to favorably interact with site one (i.e., the substrate binding site). This result agreed with the data from the literature that proposed a non-competitive inhibition for GSSK [42]. This ultimately supported the reliability of the methodology proposed to investigate the possible mechanism of inhibition. 

Then, the interaction of CSSL and PGG within the sites identified for the orthologue sequences CSSV and PGGP was calculated to investigate whether the sequence modifications could affect the capability to interact with the enzyme. Specifically, the interaction of CSSL was calculated at site one, while the interaction of PGG was calculated at site one and three. As shown in Table 3, both peptides recorded relatively high scores pointing to their capability to favorably interact with the respective site of interaction. Of note, PGG recorded scores lower than that of PGGP, possibly suggesting the formation of a weaker interaction. However, the lower number of atoms of PGG (31 atoms) compared to PGGP (45 atoms) could also have had a role to determine the lower score considering that each single-atom contribution concurs to the overall score. As shown in Figure 2, molecular dynamics confirmed the capability of CSSL and PGG to stably interact with the respective binding sites. Therefore, these results suggested that the V > L substitution found in CSSL and the removal of the Proline at the C-terminus of PGG are not likely to significantly prevent the interaction with the pancreatic alpha amylase. On this basis, CSSL and PGG may have alpha amylase inhibitory properties and they could be considered strong candidates for further dedicated analysis. Importantly, CSSL and PGG were found in the sequence of AFA’s C-phycocyanin beta subunit, which is a well-known abundant protein with orthologs already described as relevant sources of bioactive peptides with potential antidiabetic properties (e.g., [44]). Taken together, these results pointed to the possible relevance of AFA as a source of peptides modulating glucose metabolism and suggested the need for specific and dedicated investigations. 

### 3.4. In Silico Protein Digestion Results

The presence of the bioactive peptide within the primary structure of a protein does not warrant the actual release during digestion or upon processing. Indeed, many factors may influence the release of specific peptides from a protein matrix [45]. In this respect, beneficial effects in living organisms should be claimed after the actual release and adsorption along the gastrointestinal tract of peptides at a significant physiological level are proved. However, several technological and industrial approaches including fermentation and enzymatic/chemical means have been developed to promote the release of desired peptides [46]. For this reason, an in silico digestion has been performed to preliminarily assess the possible release of ELS, CSSL and PGG in the proteins they were found in (see above) upon gastrointestinal digestion or food processing. Specifically, we pipelined the RPG (v. 1.2.4) tool [33], which provides 42 enzymatic and chemical means to hydrolyze protein based on their primary sequence, to an in-house python script (see Section 2.3) to check all their possible paired combinations. A series of coupled enzymes/chemicals able to release all the peptides except PGG has been found (Table 4). 

The inability to release PGG was likely due to the presence of a Pro residue that inherently confers a certain resistance to hydrolysis. Concerning the release of ELS and CSSL, some of the effective conditions involved enzymes already used in the food industry such as papain, bromelain, ficin and thermolysin [47]. This result pointed to the likely release of both sequences upon food/protein processing suggesting the possible relevance of ELS- and CSSL-containing proteins as an effective peptide source to investigate further. Starting from these results, the development of fit-for-purpose processes may be envisaged for the future to maximize the yield of release of such peptides from algal proteins.

As a general comment, RPG was used in the “concurrent” mode simulating the simultaneous use of proteases and other chemical means [33]. This allowed a simulating environment closer to a real-world condition than other benchmark software making an “hysteretic” digestion where each protease/chemical recursively processes the digestion product of a previous combination. This warrants the accessibility of a wider number of cleavage sites providing a more reliable estimate of the peptide mixtures generated in the output [33]. Nevertheless, protease activities are approximated at their optimum in terms of pH, ionic strength and temperature, making the peptide release hard to estimate from a quantitative point of view. However, the in silico digestion performed in this study provided important information to further design effective biochemical/chemical strategies to release the desired peptides.

## 4. Conclusions 

This work presented a computational workflow based on an in-depth literature search collecting publicly available sequences with inhibitory activity against the pancreatic alpha amylase to: (i) identify novel possible protein sources of already described inhibitory sequences by means of bioinformatics; and (ii) identify novel active sequences with a high inhibitory potential for further analysis using a molecular modeling approach. The study focused on algae and cyanobacteria, chosen as case study to describe the relevance of bioinformatics and molecular modeling to investigate emerging and scarcely characterized organisms meant to be used in the food and feed production chain. The pipeline also included the calculation of peptide release upon hydrolysis for a more relevant and reliable evaluation of identified algal and cyanobacteria proteins as bioactive peptides source. Concerning the reliability of the computational workflow and methods used, the pipeline itself has been widely validated in previous works succeeding to identify bioactive peptides for a variety of pharmacologically relevant targets [1,43,48]. In addition, its fit-for-purpose validation and accuracy to alpha amylase inhibition was further confirmed based on the match between the results of reference peptides included in the study and the experimental data available in the literature.

The study described that ELS, which is among the most potent inhibitory short peptides of alpha amylase described so far, is present in proteins of *C. vulgaris* and AFA, pointing to their possible relevant source of previously characterized bioactive sequences. Moreover, CSSL and PGG have been identified within the annotated proteome of AFA and described for the first time, to the best of our knowledge, as possible alpha amylase inhibitory peptides. Their occurrence in the AFA’s C-phycocyanin beta subunit, which is a well-known very abundant protein, could suggest a high degree of relevance ultimately prioritizing those sequences and AFA for further dedicated investigations. However, cyanobacteria have been also associated with toxicological risks [49] and the use of whole harvested organisms in food and feed production should be advisably considered after a careful risk–benefit assessment. 

Overall, this study: (i) highlighted the presence of alpha amylase inhibitory sequences, including two potential peptides never described before, in the proteome of AFA and *C. vulgaris*; (ii) identified with precision the bearing proteins, which were deemed relevant from a food production standpoint; (iii) pointed out the possible role of AFA and *C. vulgaris* as a source of bioactive peptides modulating glucose metabolism and disposability, shedding light on the likely mechanisms underlying the beneficial effects of algae previously reported; and (iv) proposed enzymes/chemicals possibly able to release desired bioactive peptides from the organisms under investigation.

Concerning possible follow-up investigation, the activity of CSSL and PGG should be further assessed from a mechanistic point of view as they may be fundamental for a rational and informed use in food/nutraceuticals production. Afterwards, the actual release of those sequences in real-world conditions (e.g., via in vitro digestive models including transepithelial adsorption and optimizing the chemical condition described above), should be assessed before moving to ex vivo or in vivo trials, which may eventually confirm their efficacy in living organisms. Finally, technological processes combining chemical and enzymatic means described in this work could be designed and further optimized to maximize the release of those sequences from the proteins that have been described.

## Figures and Tables

**Table 3:** Docking scores of CSSV, PGGP, GGSK, CSSL and PGG.

Peptide	Site 1 (Substrate Binding Site)	Site 2	Site 3	Site 4
CSSV	70 *	70 *	64	65
GGSK	67	72 *	65	72 *
PGGP	63 *	54	64 *	61
CSSL	70 *	np	np	np
PGG	51 *	np	53 *	np

Note: * indicates the binding poses investigated via molecular dynamic simulations; “np” stands for “docking study not performed”.

**Table 4:** Released peptides after the in silico digestion procedure using RPG software.

Released Peptide	UniProt AC	Condition 1/Condition 2
ELS	A0A0B0QJN8	Endoproteinase Arg-C/Ficin
		Endoproteinase Arg-C/Thermolysin
		Clostripain/Ficin
		Clostripain/Thermolysin
		Ficin/Papain
		Ficin/Trypsin
		Papain/Thermolysin
		Thermolysin/Trypsin
	P32978	Ficin/Formic Acid
	P56290	Neutrophil-elastase/Ficin
	P56369	Neutrophil-elastase/Ficin
	P56298	Bromelain/Ficin
		Bromelain/Thermolysin
		Neutrophil-elastase/Ficin
		Neutrophil-elastase/Thermolysin
	P56318	BNPS-Skatole ^a^/Ficin
		BNPS-Skatole ^a^/Thermolysin
		Chymotrypsin/Ficin
		Chymotrypsin/Thermolysin
		Iodosobenzoic acid/Ficin
		Iodosobenzoic acid/Thermolysin
CSSL	P85869	Endoproetinase Asp-N/Chymotrypsin
		Endoproetinase Asp-N/Proteinase-K
		Chymotrypsin/Formic acid
		Chymotrypsin/Endoproteinase Glu-C
		Chymotrypsin/NTCB ^b^
		Formic acid/Proteinase-K
		Endoproteinase Glu-C/Proteinase-K
		NTCB ^b^/Proteinase-K

Note: ^a^ [2-(2-nitrophenyl)-3-methyl-3-bromoindolenine]; ^b^ 2-nitro-5-thiocyanatobenzoic acid. The software was used in the “concurrent mode” considering the two conditions simultaneous and not sequential.

## Data Availability

Data and “in-house” scripts developed and used in this work are available upon request.

## Supplementary Materials

The following supporting information can be downloaded at: https://www.mdpi.com/article/10.3390/nu14214680/s1, Table S1: List of papers containing alpha amylase inhibitory peptides retrieved from the literature; Table S2: Example of table generated after parsing the performed sequence alignment; Table S3: UniProt accession code of reviewed proteins analyzed. List of references cited in Supplementary Materials: [50,51,52,53,54].
