# Supplementary material for: An In Silico Framework to Mine Bioactive Peptides from Annotated Proteomes: A Case Study on Pancreatic Alpha Amylase Inhibitory Peptides from Algae and Cyanobacteria
Source: Nutrients. 2022 Nov 4;14(21):4680. doi: 10.3390/nu14214680 (PMC9658718; doi:10.3390/nu14214680)
Supplement: Supplementary file 1 [file nutrients-14-04680-s001.zip › nutrients-1940935-supplementary.pdf]

Supplementary material

An In Silico Framework to Mine Bioactive Peptides from Annotated Proteomes: A Case Study on Pancreatic Alpha Amylase Inhibitory Peptides from Algae and Cyanobacteria

Table S1. List of papers containing alpha amylase inhibitory peptides retrieved from the literature

First author	Year of publication	DOI
Ochiai *et al.* [50]	2012	https://doi.org/10.1271/bbb.110963
Yu *et al.* [51]	2012	https://doi.org/10.1016/j.foodchem.2012.06.088
Ngoh *et al.* [52]	2016	https://doi.org/10.1016/j.enzmictec.2016.04.001
Ngoh *et al.* [53]	2017	https://doi.org/10.1016/j.jff.2017.06.037
Ramadhan *et al.* [41]	2017	https://doi.org/10.1080/10942912.2017.1354885
Admassu *et al.* [42]	2018	https://doi.org/10.1021/acs.jafc.8b00960
Mudgil *et al.* [54]	2018	https://doi.org/10.1016/j.foodchem.2018.03.082
Awosika *et al.* [39]	2019	https://doi.org/10.1111/ijfs.14087
Mudgil *et al.* [40]	2021	https://doi.org/10.1016/j.foodchem.2021.129374

Table S2. Model table obtained via an in-house python script after parsing the computed alignments. The randomly selected results (n=14) are here reported to give a glance of the whole table

Peptide Source [1]	Peptide Length	Protein AC [2]	Identity % [3]	Coverage % [4]	Similarity % [5]
SAAP-CHINESE GIANT SALAMANDER	4	A0A087SCK4	100	100	100
MPSKPPLL-CAMEL MILK PROTEIN	8	A0A087SNZ9	100	63	100
KLPGF-OVALBUMIN	5	A0A3M7L4J3	50	80	100
YSFR-CHINESE GIANT SALAMANDER	4	A0A087S9R0	67	75	100
KDLWDDFKGL-CAMEL MILK PROTEIN	10	A0A1D2A5H4	100	50	100
CSSV-CHINESE GIANT SALAMANDER	4	A0A3M7KXG9	100	100	100
PPHMGGP-PINTO BEAN	7	A0A1B7X551	40	71	80
PGGP-CHINESE GIANT SALAMANDER	4	A0A1D1ZMS4	100	100	100
SAAP-CHINESE GIANT SALAMANDER	4	A0A0B0QPM2	100	100	100
EAGVD-OVALBUMIN	5	A0A1B7X1X6	100	100	100
PLPLHMLP-PINTO BEAN	8	A0A087SNH2	100	100	100
LGGGN-CHINESE GIANT SALAMANDER	5	A0A087S9I4	60	100	80
ELS-RED SEAWEED	3	A0A1B7WWG9	100	67	100
PPHMLP-PINTO BEAN	6	A0A3M7KYS1	100	33	100

[1] Indicates the active sequence (e.g. SAAP) and the source organism where the peptide was described and studied for the first time (e.g. Chinese Giant Salamander)

[2] Indicates the UniProt accession code (AC) of the algae protein where the active peptide or a similar sequence (according to the Smith-Waterman algorithm; see Section 2) has been found

[3] Indicates the identity percentage between the active peptide described in previous studies and algae protein fragment it has been aligned to

[4] Indicates the sequence coverage percentage. It is given by the ratio of the number of residues of the active peptide aligned on the algae protein and the total number of residues composing the active peptide.

[5] Indicates the sequence similarity percentage according to the Smith-Waterman algorithm; see Section 2 for further details

Table S3. UniProt accession code of reviewed proteins analysed in this study

**Algae**	**UniProt codes**
AFA	A0A0B0QJR1, A0A0B0QJN8, P85869, P00116, P00244, P56151, P85868
A. protothecoides	Q6VQA9, Q6VQA8, Q7YKW4, P13347, P13348, Q9XFY6
C. vulgaris	P56318, Q9ZRJ4, P12466, P56341, P56319, P56291, P56342, P56302, P56304, P56293, P56301, P56294, P56308, Q01170, P56303, P56344, P56297, P56316, P56300, P12465, P56323, P32978, P56317, P56321, P56309, P56310, P56362, P56354, O20163, P56306, P56361, P56359, P56352, P56296, P56292, P56295, P56315, P56305, P56350, P56346, P56339, P56325, P56348, P56340, P56322, P56368, P56298, P58214, P56338, P32975, P56299, P56365, P56355, P56357, P56356, P56327, P32976, P56351, P56358, P56366, P56363, P56360, P56367, P56353, P56324, O20143, P56307, P56364, P56312, P56370, O20120, O20133, P56328, O20162, P56326, P56345, P56314, O20142, O20159, O20130, O20173, P56311, P56313, O20118, P56290, P56349, P56347, P56369, P32979, P56343
